# Identification of novel candidate biomarkers and immune infiltration in polycystic ovary syndrome

**DOI:** 10.1186/s13048-022-01013-0

**Published:** 2022-07-06

**Authors:** Zhijing Na, Wen Guo, Jiahui Song, Di Feng, Yuanyuan Fang, Da Li

**Affiliations:** 1 Center of Reproductive Medicine, Shengjing Hospital of China Medical University, No.36, SanHao Street, Shenyang, 110004 China; 2 Department of Neurology, The First Hospital of China Medical University, Shenyang, 110002 China; 3 Education Center for Clinical Skills Practice of China Medical University, Shenyang, 110122 China; 4 Key Laboratory of Reproductive Dysfunction Diseases and Fertility Remodeling of Liaoning Province, Shenyang, 110122 China

**Keywords:** Polycystic ovary syndrome, Biomarkers, Immune infiltration, Machine learning algorithm, CIBERSORT

## Abstract

**Background:**

In this study, we aimed to identify novel biomarkers for polycystic ovary syndrome (PCOS) and analyze their potential roles in immune infiltration during PCOS pathogenesis.

**Methods:**

Five datasets, namely *GSE137684*, *GSE80432*, *GSE114419*, *GSE138518*, and *GSE155489*, were obtained from the Gene Expression Omnibus database. Differentially expressed genes (DEGs) were selected from the train datasets. The least absolute shrinkage and selection operator logistic regression model and support vector machine-recursive feature elimination algorithm were combined to screen potential biomarkers. The test datasets validated the expression levels of these biomarkers, and the area under the curve (AUC) was calculated to analyze their diagnostic value. Quantitative real-time PCR was conducted to verify biomarkers’ expression in clinical samples. CIBERSORT was used to assess differential immune infiltration, and the correlations of biomarkers with infiltrating immune cells were evaluated.

**Results:**

Herein, 1265 DEGs were identified between PCOS and control groups. The gene sets related to immune response and adaptive immune response were differentially activated in PCOS. The two diagnostic biomarkers of PCOS identified by us were HD domain containing 3 (HDDC3) and syndecan 2 (SDC2; AUC, 0.918 and 0.816, respectively). The validation of hub biomarkers in clinical samples using RT-qPCR was consistent with bioinformatics results. Immune infiltration analysis indicated that decreased activated mast cells (*P* = 0.033) and increased eosinophils (*P* = 0.040) may be a part of the pathogenesis of PCOS. HDDC3 was positively correlated with T regulatory cells (*P* = 0.0064), activated mast cells (*P* = 0.014), and monocytes (*P* = 0.024) but negatively correlated with activated memory CD4 T cells (*P* = 0.016) in PCOS. In addition, SDC2 was positively correlated with activated mast cells (*P* = 0.0021), plasma cells (*P* = 0.0051), and M2 macrophages (*P* = 0.038) but negatively correlated with eosinophils (*P* = 0.01) and neutrophils (*P* = 0.031) in PCOS.

**Conclusion:**

HDDC3 and SDC2 can serve as candidate biomarkers of PCOS and provide new insights into the molecular mechanisms of immune regulation in PCOS.

**Supplementary Information:**

The online version contains supplementary material available at 10.1186/s13048-022-01013-0.

## Introduction

Polycystic ovary syndrome (PCOS) is a common endocrine disorder affecting 5–15% of women of childbearing age, and it presents with chronic anovulation as the main feature [1, 2]. Before ovulation, granulosa cells (GCs) act on oocyte growth, differentiation, and meiosis [3]. GCs co-operate with neighboring immune cells in the physiological state to produce paracrine mediators, thus promoting ovulation [4]. Previous studies have indicated that PCOS presents with a chronic inflammatory status, and being the primary source of inflammatory cytokines, immune cells play a role in PCOS [5, 6]. The impaired regulation of GCs and immune cells under the pathological state of PCOS may accelerate anovulation [7]. However, the combined regulatory mechanisms of GCs and immune cells in the progression of PCOS have not been fully elucidated.

Support vector machine-recursive feature elimination (SVM-RFE), a sub-method of machine learning, offers an advantage in explaining the strength and direction of interactions between predictors and outcomes by RFE of non-linear kernels [8]. CIBERSORT, a gene expression-based deconvolution algorithm, assesses immune cell infiltration signatures [9]. However, to our knowledge, SVM-RFE and CIBERSORT algorithms have not been used to select potential biomarkers and predict differential infiltrating immune cells in PCOS.

In this study, we aimed to screen novel biomarkers in GCs related to PCOS using a machine learning strategy. Furthermore, to better understand the molecular mechanisms of immune regulation in PCOS, we evaluated immune cell infiltration using the CIBERSORT algorithm and analyzed the relationship of the biomarkers with the infiltrated immune cells in PCOS.

## Materials and methods

### Data selection and preprocessing

We searched the Gene Expression Omnibus (GEO) database using the following keywords: (“PCOS” OR “polycystic ovary syndrome” OR “Stein-Leventhal syndrome” OR “sclerocystic ovarian degeneration” OR “sclerocystic ovaries” OR “sclerocystic ovary syndrome”) AND (“granulosa cells” OR “GC” OR “cumulus cells” OR “granulosa cumulus cells”) AND (“human” OR “homo sapiens”). Searches within GEO were filtered by “expression profiling by array” and “expression profiling by high-throughput sequencing.” Seven datasets were retrieved from the search, and two datasets were excluded due to their lack of gene annotation. The remaining five datasets included three microarray datasets and two RNA-sequencing (RNA-seq) datasets. Datasets of the same sequencing type were analyzed together. The three microarray datasets, including *GSE137684*, *GSE80432*, and *GSE114419*, contained GC samples from 19 PCOS patients and 15 control subjects. The two RNA-seq datasets, including *GSE138518* and *GSE155489*, contained GC samples from seven PCOS patients and seven control subjects. The three microarray datasets were considered as the train datasets due to their larger sample sizes compared to those of the two RNA-seq datasets, and the two RNA-seq datasets were then used for test validation. The R package’s “save and standardization” algorithms were used to preprocess batch effect removal on the train and test datasets.

### Identification of differentially expressed genes (DEGs)

The R package *limma* was used to analyze DEGs in GCs between PCOS patients and control subjects, and genes with a *P*-value of <0.05 and log fold change >1 were selected as DEGs. Heatmaps and volcano plots were made using the R packages *pheatmap* and *ggplot2* to further visualize the up- and down-regulated DEGs, respectively.

### Functional enrichment analyses

Gene ontology (GO) functional analyses of DEGs were performed to evaluate their biological processes (BP), cellular components (CC), and molecular functions (MF) using the R package *clusterProfiler*. Moreover, Kyoto encyclopedia of genes and genomes (KEGG) pathway analyses of DEGs were conducted using the same R package. Disease ontology (DO) enrichment analysis was performed through the R package *DOSE*. Gene set enrichment analysis (GSEA) was performed using GSEA v4.1.0 to further assess the related function enrichments of DEGs. KEGG maps of biological functions associated with a *P*-value of <0.05 were determined to be significantly enriched, and the results of GSEA with a *P*-value of <0.05 were considered significant. In addition, the false discovery rate-adjusted *P*-value was defined as the *Q*-value, and the cutoff criteria for GO and DO analyses were set as a *Q*-value of <0.05.

### A comprehensive strategy for screening candidate biomarkers

We used a comprehensive selection method based on the least absolute shrinkage and selection operator (LASSO) logistic regression model and SVM-RFE algorithm to analyze PCOS-related biomarkers. The LASSO logistic regression model selects biomarkers using LASSO in the R package *glmnet* [10]. SVM-RFE is an iterative approach combining linear support vector machines with feature selection and backward elimination, which is implemented with the R packages *e1071*, *kernlab*, and *caret* [8]. To further assess the diagnostic ability of candidate biomarkers, the receiver operating characteristic (ROC) curve and calculations of its area under the curve (AUC), accuracy, sensitivity, and specificity were performed using the R package *pROC*. *P* < 0.05 indicated significant differences.

### Immune infiltration estimations via CIBERSORT

The CIBERSORT (https://cibersort.stanford.edu/) calculation algorithm was used to predict the differential abundance of immune infiltrating cells between PCOS patients and control subjects. Correlation heatmaps and violin plots were prepared to visualize the results of CIBERSORT using the R packages *corrplot* and *ggplot2*, respectively [9].

### Interaction analysis of selected biomarkers

Spearman correlation coefficients calculated using the R statistical package evaluated the relationships between hub biomarkers and infiltrating immune cells. The interaction results were visualized through the R package *ggplot2*.

### Study subjects

In this study, we recruited 10 women who underwent *in vitro* fertilization and embryo transfer (IVF-ET) at the Reproductive Center of Shengjing Hospital, China Medical University, Shenyang, between August 2021 and October 2021. According to the Rotterdam criteria, five patients were diagnosed as having PCOS, and five control women were either infertile due to fallopian tube obstruction or could not conceive due to their husband’s subfertility. Subjects were excluded if they had a history of diabetes, chronic kidney disease, chronic metabolic disease, or endometriosis. The Health Research Ethics Board of the Shengjing Hospital approved the study.

### Sample acquisition

Individual subjects were administered recombinant follicle-stimulating hormone (FSH, 150–187.5 IU; Gonal-f, Follitropin Alfa, Serono) and a gonadotropin-releasing hormone agonist for ovarian hyperstimulation. The subjects were given 250 μg human chorionic gonadotropin (hCG, Profasi; Serono) if two follicles had a diameter of 18 mm and the serum contents of E2 were 300 pg/mL per predominant follicle. Then, 36 h after hCG administration, follicular fluid (2 mL) was extracted from the predominant follicles through a vaginal puncture under ultrasound echo guidance. The collected follicular fluid was immediately centrifuged at 700 *g* for 5 min. The precipitates were suspended and gently layered onto 3 mL of 50% lymphocyte separation medium (Biosharp, Anhui, China). After centrifugation at 700 *g* for 10 min, GCs were washed twice with phosphate-buffered saline (Biosharp), collected in TRIzol® reagent (Thermo-Fisher Scientific, USA), and stored at −80 °C until RNA extraction.

### Quantitative real-time PCR (RT-qPCR)

Total RNA in GC samples was extracted using the TRIzol® reagent (Thermo-Fisher Scientific, USA) and reverse transcribed into cDNA according to the instructions of the PrimeScript™ RT Reagent Kit and gDNA Eraser (TaKaRa, China). The reaction was performed using a thermal cycler at 42 °C for 2 min, 37 °C for 15 min, and 85 °C for 15 s. RNA transcript levels were quantified by RT-qPCR using the TaKaRa-SYBR® Premix Ex TaqTM II (TaKaRa, Japan) and specific primers (Supplementary Table 2) performed on the ABI ViiA 7 Real-time PCR Platform (Applied Biosystems, USA) according to the manufacturer’s protocol. The qPCR reaction comprised 10-μL SYBR Premix Ex Taq II (2×), 0.8-μL PCR forward primer (10 μM), 0.8-μL PCR reverse primer (10 μM), 6.4-μL RNase-free ddH2O, and 2-μL cDNA (Takara, Japan). PCR amplification was performed as follows: predegeneration at 95 °C for 30 s, 40 cycles of degeneration at 95 °C for 5 s, and annealing at 60 °C for 30 s. GAPDH served as an internal control, and 2-^ΔΔCt^ was used for data analysis of relative gene expression.

## Results

### Identification of DEGs

**Fig. 1:**
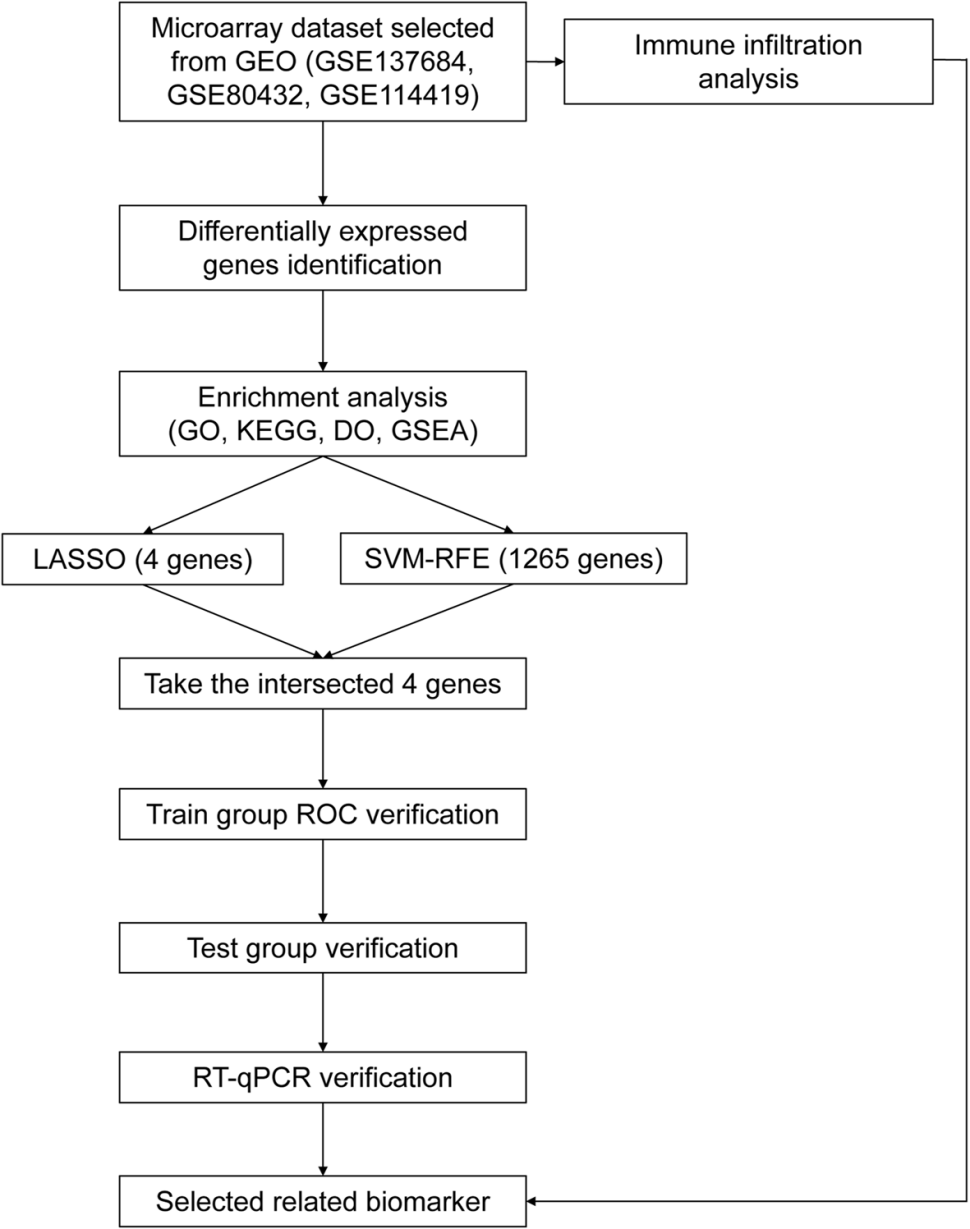
Workflow chart of data generation and analysis

**Fig. 2:**
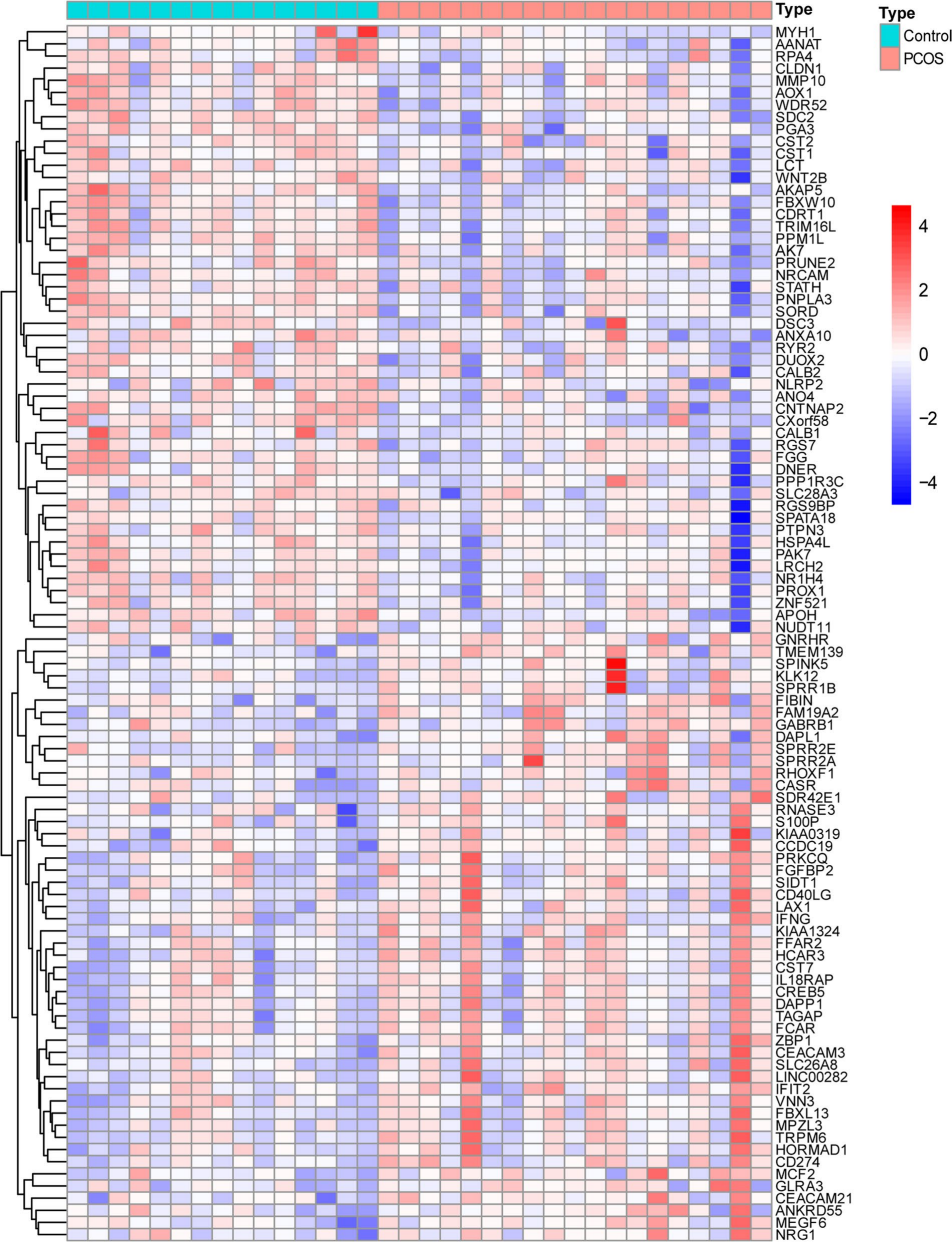
The expression characteristics of genes in granulosa cells of the PCOS patients and control subjects. The heatmap presents the overall expression with *P* < 0.05 in the PCOS and control groups

Figure 1 shows the overall workflow of this study. DEGs were analyzed in the three train datasets *GSE137684*, *GSE80432*, and *GSE114419*, including GC samples of 19 PCOS patients and 15 control subjects. A total of 1265 DEGs, comprising 387 upregulated genes and 878 downregulated genes, were screened in the PCOS group, compared with the control group. Heatmaps were subsequently drawn to display these DEGs (Fig. 2).

### Enrichment analysis

**Fig. 3:**
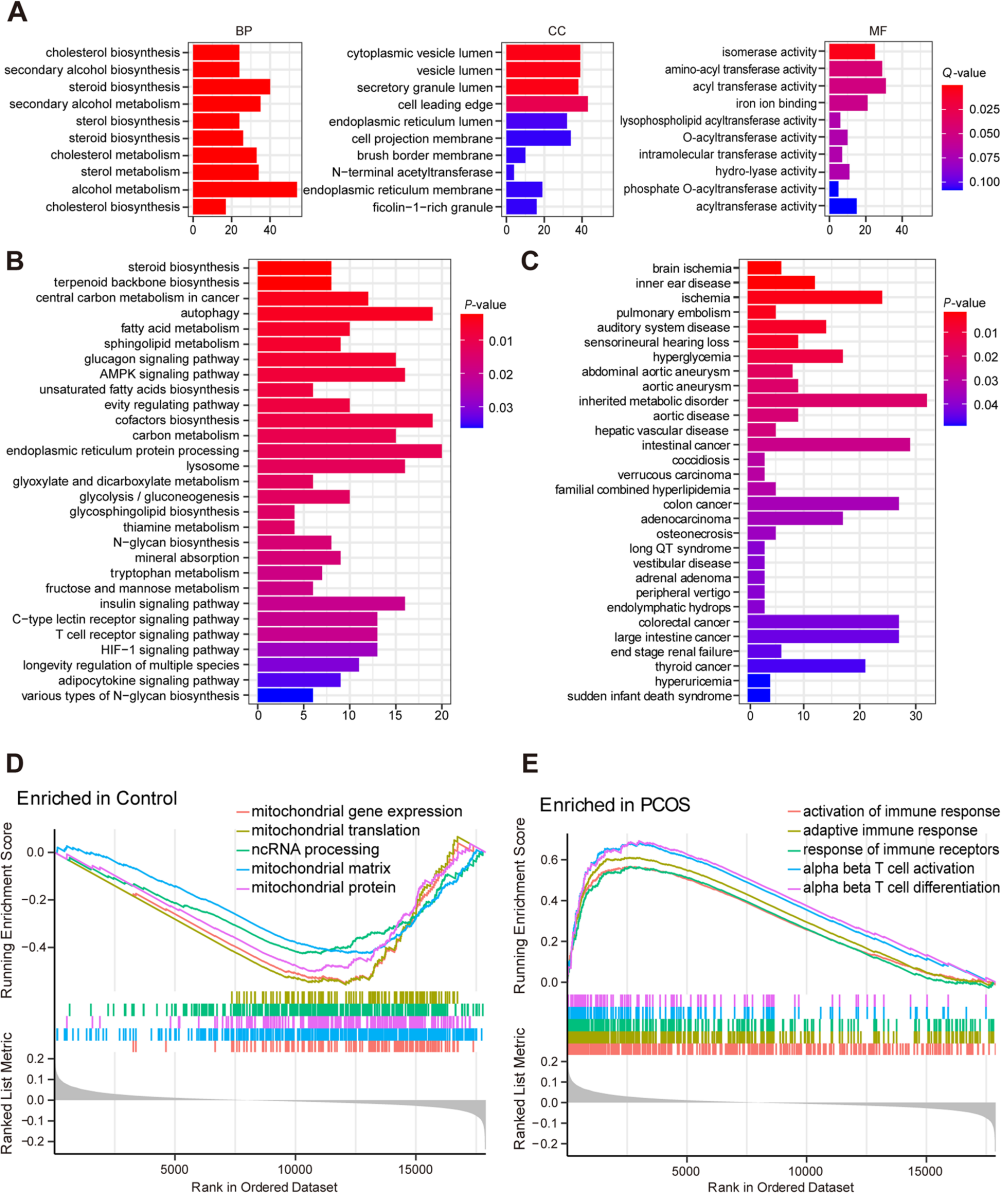
Functional enrichment analyses of DEGs. **A** GO analyses were conducted to predict the potential functions of DEGs between the PCOS and control groups, including CC, MF, and BP. **B** KEGG potential pathways regarding DEGs between the PCOS and control groups were evaluated. **C** DO analyses were conducted to predict the diseases potentially related to DEGs between the PCOS and control groups. **D** GSEA showed the top five signal pathways that were most likely expressed in the control group. **E** GSEA showed the top five signal pathways that were the most related to the PCOS group. DEGs, differentially expressed genes; GO, gene ontology; PCOS, polycystic ovary syndrome; CC, cellular component; MF, molecular function; BP, biological process; KEGG, Kyoto encyclopedia of genes and genomes; DO, disease ontology; GSEA, gene set enrichment analysis

We performed GO and KEGG pathway enrichment analyses on DEGs using the R package *clusterProfiler*. The top 10 BP, CC, and MF were identified according to the screening criteria of *Q*-value being <0.05, with the alcohol metabolic process, steroid biosynthetic process, cytoplasmic vesicle lumen, and isomerase activity being the enriched functions in the GC samples of PCOS patients (Fig. 3A). KEGG analysis results revealed several enriched pathways in PCOS, including autophagy, AMPK signaling pathway, biosynthesis of cofactors, and protein processing in the endoplasmic reticulum (Fig. 3B). The enriched related diseases of DEGs were obtained with a *Q*-value of <0.05 via DO enrichment analysis (Fig. 3C). Additionally, GSEA analysis indicated close relationships of enriched gene sets with the activation of immune response, adaptive immune response, and alpha-beta T cell activation and differentiation in the PCOS group (Fig. 3D, E).

### Candidate biomarkers screening with machine learning strategy

**Fig. 4:**
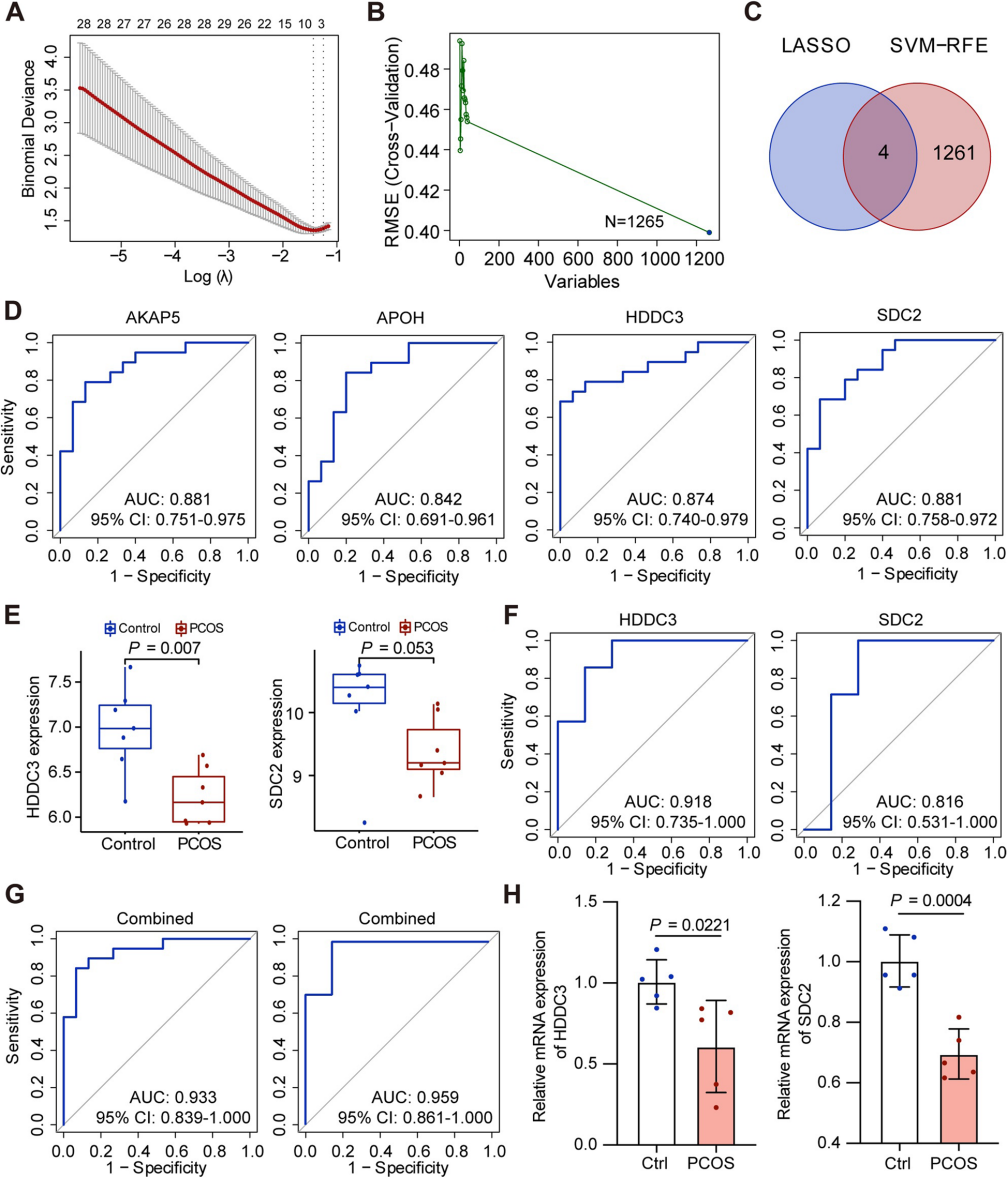
Identification of the hub biomarkers in PCOS via a comprehensive strategy. **A** The LASSO logistic regression model was used to retain the most predictive features. **B** Biomarkers were screened based on the SVM-RFE algorithm. **C** The Venn diagram showed the intersection of biomarkers obtained by the LASSO logistic regression model and SVM-RFE algorithm. **D** The ROC curves of AKAP5, APOH, HDDC3, and SDC2 in the train group. **E** The differential expressions of HDDC3 and SDC2 in the test group. **F** The ROC curves of HDDC3 and SDC2 in the test group. **G** The ROC curves of combining HDDC3 and SDC2 in the train and test groups. **H** Relative quantification of the hub genes HDDC3 and SDC2 based on RT-qPCR results. PCOS, polycystic ovary syndrome; LASSO, least absolute shrinkage and selection operator; SVM-RFE, support vector machine-recursive feature elimination; ROC, receiver operating characteristic; AKAP5, A-kinase anchoring protein 5; APOH, apolipoprotein H; HDDC3, HD domain containing 3; SDC2, syndecan 2

Two gene sets of potential PCOS-related biomarkers from DEGs were initially identified using the LASSO logistic regression model and SVM-RFE algorithm, respectively (Fig. 4A, B). As shown in a Venn diagram (Fig. 4C), examination of these two gene sets helped us identify four characteristic PCOS-related biomarkers, namely A-kinase anchoring protein 5 (AKAP5), apolipoprotein H (APOH), HD domain containing 3 (HDDC3), and syndecan 2 (SDC2).

### Predicted performance of hub biomarkers

To further identify the hub biomarkers from the four candidate biomarkers, ROC analysis was performed for the train datasets, and all of these four biomarkers had an AUC of >0.8 fpr predicing PCOS (Fig. 4D). Moreover, two datasets (*GSE138518* and *GSE155489*) were used to validate the expressions of these four potential biomarkers. Differential expression of two genes, namely HDDC3 and SDC2, was verified in the test group (Fig. 4E). HDDC3 and SDC2 were expressed significantly lower in the GCs of PCOS patients than in those of control subjects. Moreover, ROC analyses of the test datasets indicated that HDDC3 and SDC2 had an AUC of 0.918 and 0.816, respectively (Fig. 4F). Using ROC curves, we also detected the cutoff, sensitivity, and specificity of HDDC3, SDC2, and their combination for the prediction of PCOS (Supplementary Table 3). Notably, the combination of HDDC3 and SDC2 yielded an AUC of >0.9 in predicting PCOS in both train and test groups (Fig. 4G). However, there was no difference in the expression of AKAP5 and APOH between PCOS patients and control subjects in the test group, and the ROC analysis results of AKAP5 and APOH in the test group were unsatisfactory (Supplementary Fig. 1). Therefore, we obtained two hub biomarkers with PCOS-specific expression, namely HDDC3 and SDC2.

### Validation of hub biomarkers in clinical samples

To verify the above bioinformatics analyses, we investigated the expression of the two hub biomarkers in GC samples from five PCOS patients and five control subjects (Fig. 4H). Using RT-qPCR, we identified that HDDC3 and SDC2 were significantly downregulated in GCs of PCOS patients compared with those of control subjects, which was consistent with our bioinformatics results.

### Immune cell infiltration analysis

**Fig. 5:**
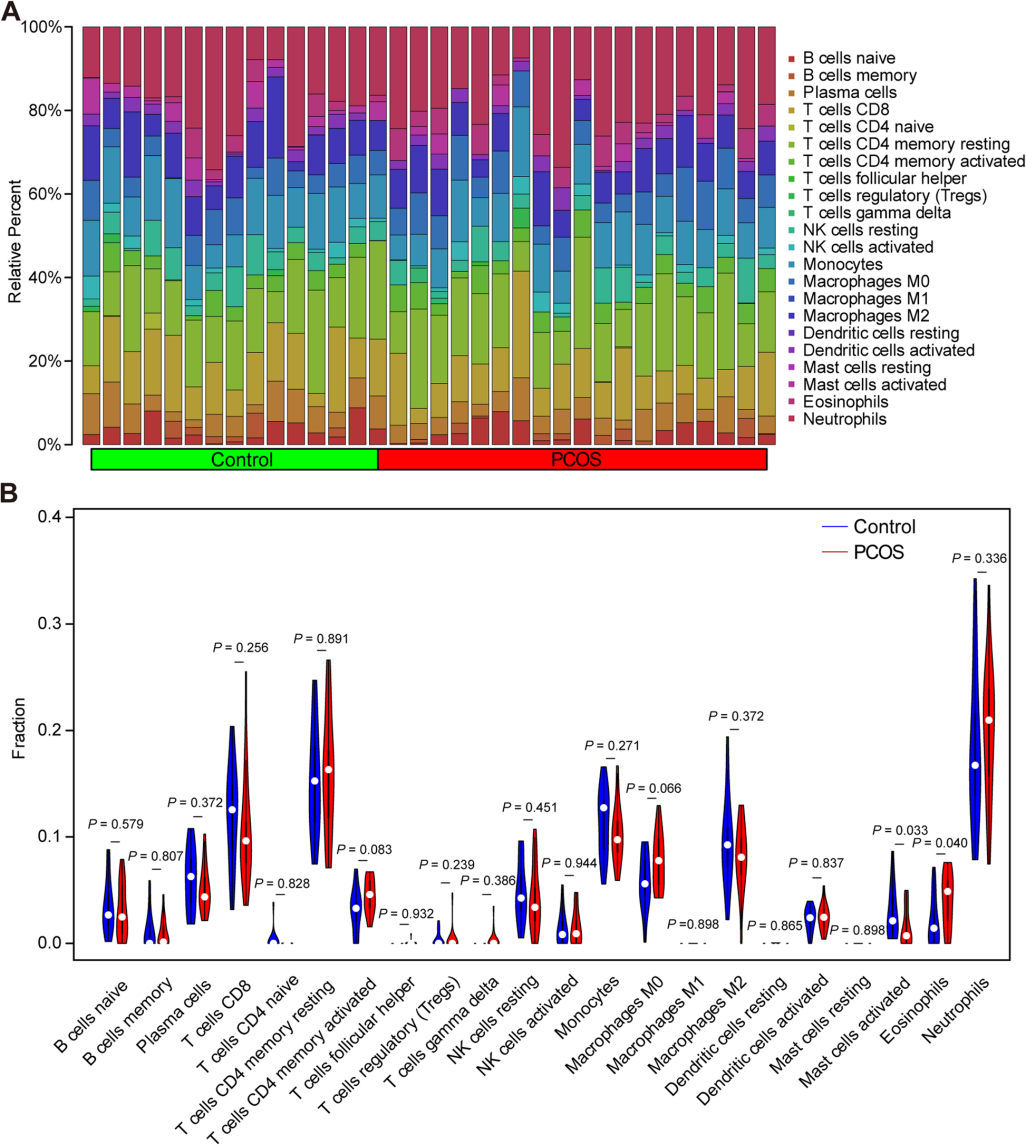
Analysis of the distribution of infiltrating immune cells. **A** Composition of immune cells in the PCOS and control groups. **B** The Violin plot visualized the differentially expressed immune cells between the PCOS (red) and control (blue) groups. PCOS, polycystic ovary syndrome

The infiltration of immune cells around GCs was predicted using the CIBERSORT algorithm, as shown in Fig. 5A and B. Compared with other immune cells, T cells CD4 memory resting and neutrophils dominated in both PCOS and control groups. A significant difference was detected in the infiltration of activated mast cells (*P* = 0.033) and eosinophils (*P* = 0.040) between groups, indicating the potential role of mast cells and eosinophils in the pathogenesis of PCOS.

### Relationship of biomarkers with infiltrating immune cells

**Fig. 6:**
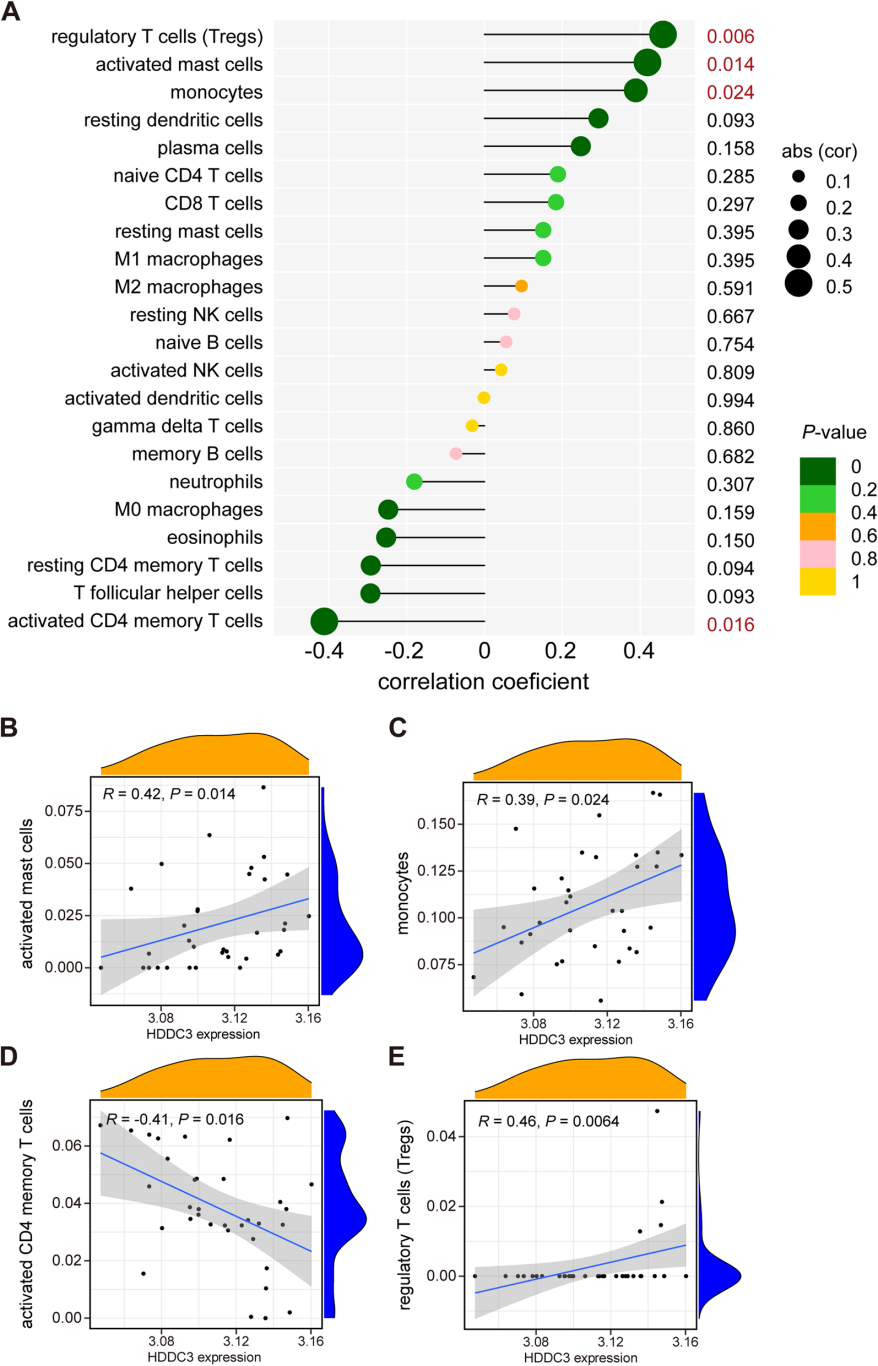
Analysis of the correlation between biomarkers and infiltrating immune cells. **A** Correlation between HDDC3 and infiltrating immune cells. **B** Correlation between HDDC3 and activated mast cells. **C** Correlation between HDDC3 and monocytes. **D** Correlation between HDDC3 and T cells CD4 memory activated. **E** Correlation between HDDC3 and T regulatory cells. HDDC3, HD domain containing 3

**Fig. 7:**
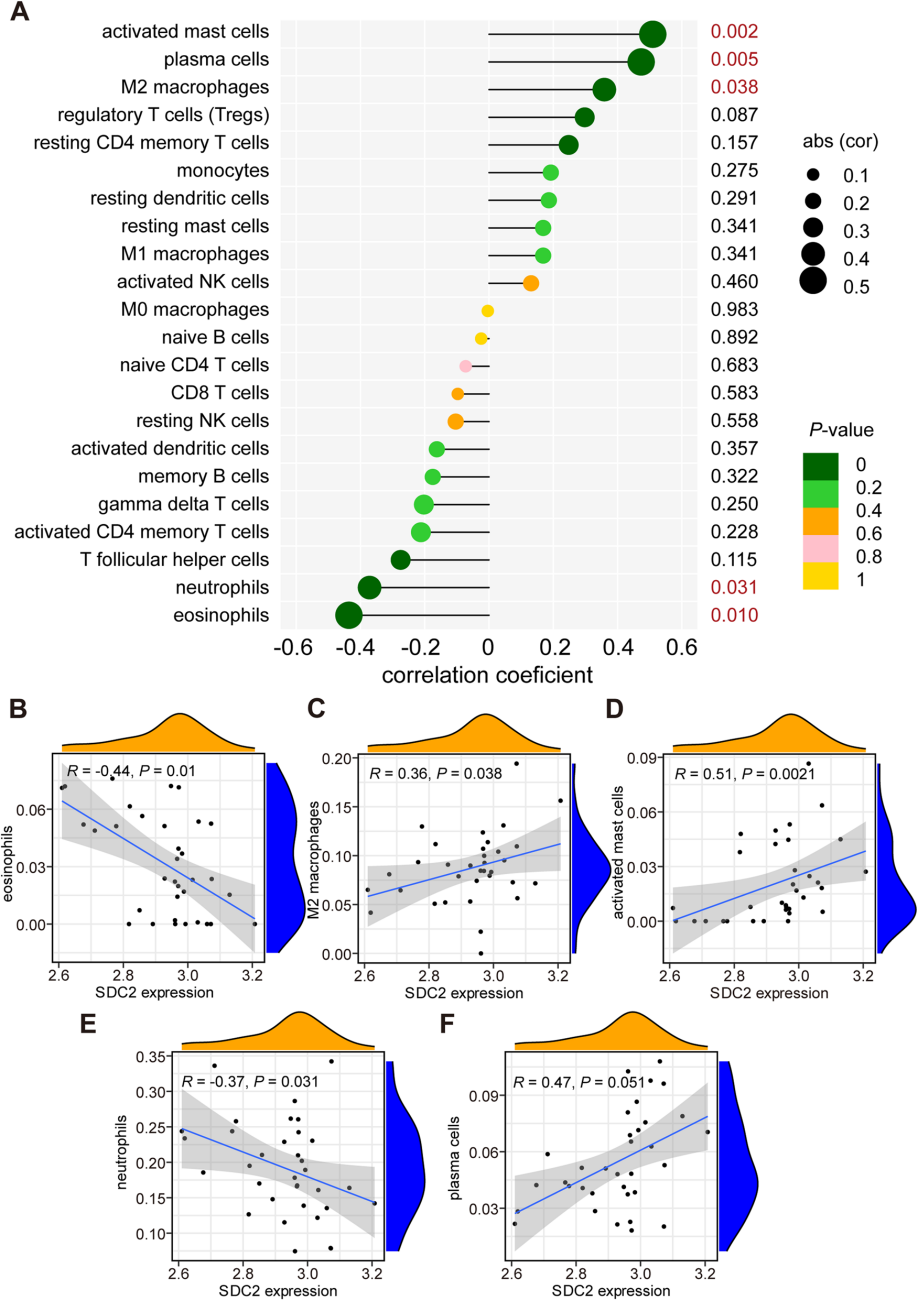
Analysis of the correlation between biomarkers and infiltrating immune cells. **A** Correlation between SDC2 and infiltrating immune cells. **B** Correlation between SDC2 and eosinophils. **C** Correlation between SDC2 and M2 macrophages. **D** Correlation between SDC2 and activated mast cells. **E** Correlation between SDC2 and neutrophils. **E** Correlation between SDC2 and plasma cells. SDC2, syndecan 2

Correlation analysis was conducted to evaluate the relationships of biomarkers with infiltrating immune cells. We identified that the hub biomarker HDDC3 was positively correlated with T regulatory cells (*R* = 0.46, *P* = 0.0064), activated mast cells (*R* = 0.42, *P* = 0.014), and monocytes (*R* = 0.39, *P* = 0.024) but negatively correlated with activated memory CD4 T cells (*R* = −0.41, *P* = 0.016; Fig. 6). Meanwhile, we identified that the hub biomarker SDC2 was positively correlated with activated mast cells (*R* = 0.51, *P* = 0.0021), plasma cells (*R* = 0.47, *P* = 0.0051), and M2 macrophages (*R* = 0.36, *P* = 0.038) but negatively correlated with eosinophils (*R* = −0.44, *P* = 0.01) and neutrophils (*R* = −0.37, *P* = 0.031; Fig. 7).

## Discussion

Recently, machine learning strategies have emerged as powerful tools to investigate the underlying relationships of high-dimensional data and select the best parameters for gene selection among all DEGs with biological significance [11]. Some studies have focused on exploring PCOS biomarkers via various machine learning algorithms [12, 13]. For example, Xie *et al*. integrated artificial neural networks and random forest algorithms to explore diagnostic markers in PCOS [12]. We herein combined the LASSO logistic regression model and SVM-RFE algorithm to determine the potential biomarkers and further verified their expression in the test datasets. The hub biomarkers identified by us in this study seem to have higher AUCs, with an average AUC of >0.8 in both train and test datasets, indicating their potential predictive abilities in PCOS.

The two hub biomarkers identified by us are HDDC3 and SDC2. HDDC3 is a cytosolic NADPH phosphatase that regulates ferroptosis [14], and ferroptosis is reportedly involved in endocrine and metabolic diseases, including PCOS [15]. SDC2 is a heparan sulfate proteoglycan that promotes TGFβ-induced immunosuppressive genes and facilitates T cell proliferation [16]. Furthermore, silencing of SDC2 inhibited macrophages’ phagocytosis of apoptotic neutrophils and consequently resulted in tissue injury [17]. This study predicted a positive relationship between SDC2 and anti-inflammatory M2 macrophages, which is consistent with previous studies. Thus, we speculated that the downregulation of SDC2 in PCOS is associated with the immune dysregulation of PCOS. To our knowledge, HDDC3 and SDC2 have not been reported in PCOS-related studies yet; however, we considered their potential roles in the metabolic and immune processes of PCOS.

PCOS presents with chronic low-grade inflammation [5, 6]. The inflammatory process activates locally infiltrating immune cells, thus leading to immune dysfunction [18]. In this study, KEGG and GSEA analyses indicated that most DEGs between PCOS and control groups were enriched in immune activation functions. CIBERSORT was used to evaluate immune infiltration in PCOS, which revealed a decreased infiltration of activated mast cells and an increased infiltration of eosinophils in PCOS. Eosinophils increase with mast cell activation [19], and it has been reported that the infiltration of eosinophils and mast cells commonly mirrors each other [20]. Our finding of opposite infiltrative patterns of mast cells and eosinophils and the potential roles of these two cells in PCOS need to be further clarified. Furthermore, the hub biomarkers HDDC3 and SDC2 were positively correlated with activated mast cells, and SDC2 was negatively correlated with eosinophils. Further experiments are necessary to define the intricate relationships of the hub biomarkers with immune infiltration in PCOS.

Recent attention has been given to the identification of diagnostic biomarkers for PCOS [21, 22]. In this study, the decreased transcript levels of HDDC3 and SDC2 were validated in granulosa cells from women with PCOS. This finding indicated that HDDC3 and SDC2 might serve as candidate biomarkers for PCOS in clinical practice; however, the clinical significance of them still requires further systematic exploration. Moreover, considering the potential relationships of HDDC3 and SDC2 with immune cells in PCOS, future studies investigating the underlying mechanisms of HDDC3 and SDC2 in PCOS will be performed.

Nevertheless, several limitations of this study also need to be considered, including a lack of existing datasets with a large sample size, data heterogeneity, and platform differences. Furthermore, it remains unclear whether HDDC3 and SDC2 contribute to the pathogenesis of immune infiltration in PCOS or are only potential biomarkers of this process.

## Conclusion

This study identified HDDC3 and SDC2 as candidate biomarkers of PCOS and evaluated their potential interactions with immune cells during the pathogenesis of PCOS. Additionally, immune cell infiltration, including the differential infiltration of eosinophils and mast cells, is involved in PCOS. Further research with a larger sample size and more predictive means of clinical applicability are needed to verify these results.

## Supplementary Information

**Additional file 1: Supplementary Figure 1.** The differential expressions of AKAP5 (A) and APOH (B) in the test group. The ROC curves of AKAP5 (C) and APOH (D) in the test group. ROC, receiver operating characteristic; AKAP5, A-kinase anchoring protein 5; APOH, apolipoprotein H.

**Additional file 2: Supplementary Table 1.** Gene expression data from the Gene Expression Omnibus (GEO) database.

**Additional file 3: Supplementary Table 2.** Primer sequences of the hub biomarkers and internal control.

**Additional file 4: Supplementary Table 3.** The performance of HDDC3, SDC2, and their combination in predicting PCOS.

## Data Availability

The datasets supporting the conclusions of this article are available in the GEO database (http://www.ncbi.nlm.nih.gov/geo).

## Abbreviations

PCOS: Polycystic ovary syndrome
DEGs: Differentially expressed genes
LASSO: Least absolute shrinkage and selection operator
SVM-RFE: Support vector machine recursive feature elimination
AUC: Area under the curve
HDDC3: HD domain containing 3
SDC2: Syndecan 2
GCs: Granulosa cells
GEO: Gene Expression Omnibus
GO: Gene ontology
BP: Biological processes
CC: Cellular components
MF: Molecular functions
KEGG: Kyoto encyclopedia of genes and genomes
DO: Disease ontology
GSEA: Gene set enrichment analysis
IVF-ET: In vitro fertilization and embryo transfer
RT-qPCR: Quantitative real-time PCR
AKAP5: Anchoring protein 5
APOH: Apolipoprotein H
