# Supplementary figures and images for: Identification of novel candidate biomarkers and immune infiltration in polycystic ovary syndrome
Source: J Ovarian Res. 2022 Jul 6;15:80. doi: 10.1186/s13048-022-01013-0 (PMC9258136; doi:10.1186/s13048-022-01013-0)

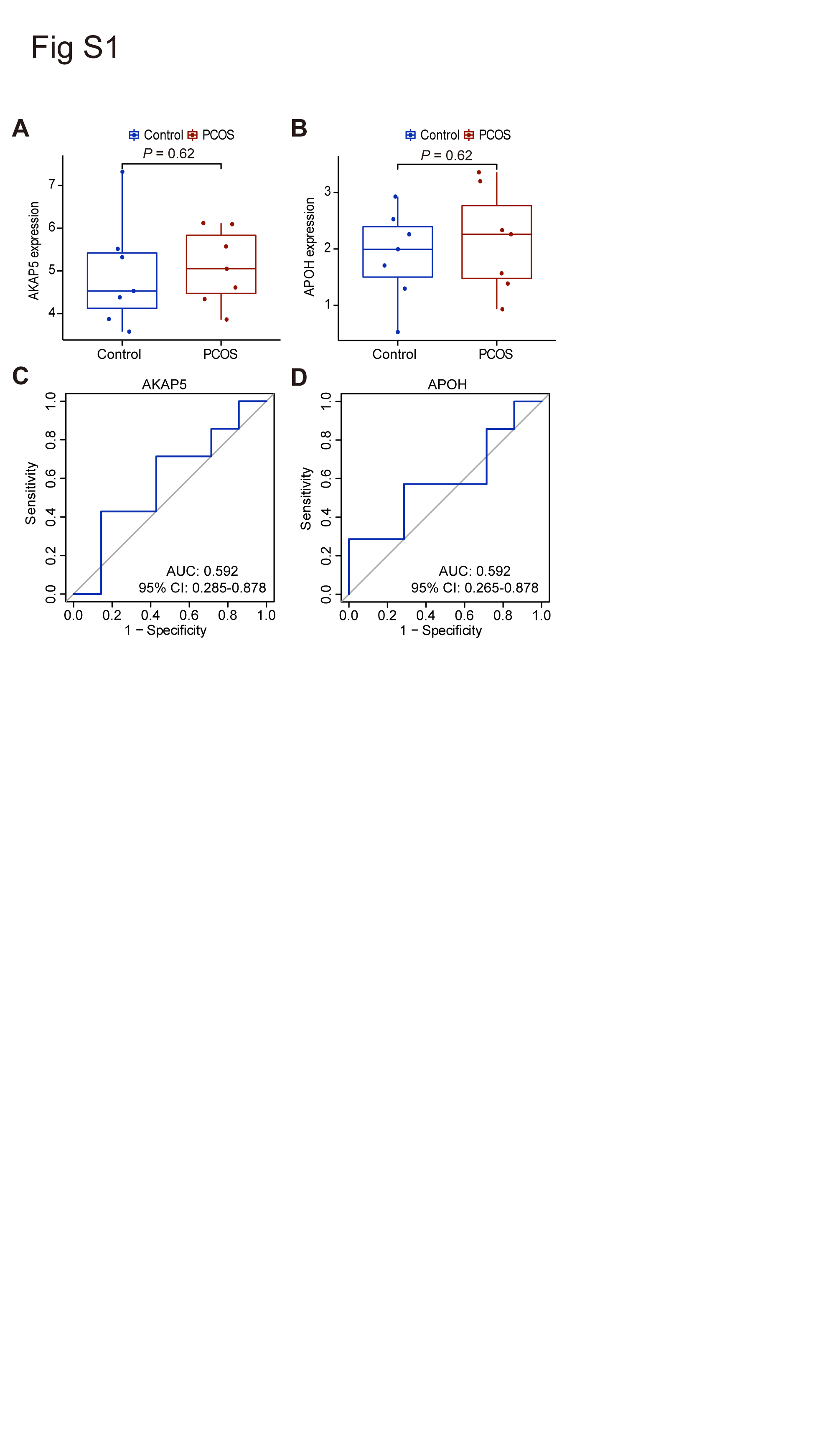

Supplement: Supplementary file 1 — Additional file 1: Supplementary Figure 1. The differential expressions of AKAP5 (A) and APOH (B) in the test group. The ROC curves of AKAP5 (C) and APOH (D) in the test group. ROC, receiver operating characteristic; AKAP5, A-kinase anchoring protein 5; APOH, apolipoprotein H. [file 13048_2022_1013_MOESM1_ESM.tif]
