# Supplementary material for: Identification of novel candidate biomarkers and immune infiltration in polycystic ovary syndrome
Source: J Ovarian Res. 2022 Jul 6;15:80. doi: 10.1186/s13048-022-01013-0 (PMC9258136; doi:10.1186/s13048-022-01013-0)
Supplement: Supplementary file 2 — Additional file 2: Supplementary Table 1. Gene expression data from the Gene Expression Omnibus (GEO) database. [file 13048_2022_1013_MOESM2_ESM.docx]

**Supplementary table 1.** Gene expression data from Gene Expression Omnibus (GEO) database.

| **Dataset ID** | **Total samples** | **Control** | **PCOS** | **Data type** | **Tissue type** | **Country** |
| --- | --- | --- | --- | --- | --- | --- |
| GSE137684 | 12 | 4 | 8 | Microarray | Granulosa cells | China |
| GSE80432 | 16 | 8 | 8 | Microarray | Granulosa cells | China |
| GSE114419 | 6 | 3 | 3 | Microarray | Granulosa cells | China |
| GSE138518 | 6 | 3 | 3 | RNA-seq | Granulosa cells | China |
| GSE155489 | 8 | 4 | 4 | RNA-seq | Granulosa cells | China |
