# Supplementary material for: Identification of novel candidate biomarkers and immune infiltration in polycystic ovary syndrome
Source: J Ovarian Res. 2022 Jul 6;15:80. doi: 10.1186/s13048-022-01013-0 (PMC9258136; doi:10.1186/s13048-022-01013-0)
Supplement: Supplementary file 3 — Additional file 3: Supplementary Table 2. Primer sequences of the hub biomarkers and internal control. [file 13048_2022_1013_MOESM3_ESM.docx]

**Supplementary table 2.** Primer sequences of the hub biomarkers and internal control.

| **Gene** | **Primer sequences** | |
| --- | --- | --- |
| HDDC3 | Forward | ACCCTGGATGAGGTGGAG |
|  | Reverse | CTACTGTGGGGCGCTTG |
| SDC2 | Forward | AGGATTGAAGTGGCTGGA |
|  | Reverse | TGGACGGCTCACAGTTTA |
| GAPDH | Forward | CCTTCCGTGTCCCCACT |
|  | Reverse | GCCTGCTTCACCACCTTC |
