# Supplementary material for: Identification of novel candidate biomarkers and immune infiltration in polycystic ovary syndrome
Source: J Ovarian Res. 2022 Jul 6;15:80. doi: 10.1186/s13048-022-01013-0 (PMC9258136; doi:10.1186/s13048-022-01013-0)
Supplement: Supplementary file 4 — Additional file 4: Supplementary Table 3. The performance of HDDC3, SDC2, and their combination in predicting PCOS. [file 13048_2022_1013_MOESM4_ESM.docx]

**Supplementary table 3.** The preformance of HDDC3, SDC2, and their combination in predicting PCOS.

|  |  | AUC | Cutoff | 95% CI | Sensitivity% | Specificity% | P-value |
| --- | --- | --- | --- | --- | --- | --- | --- |
| Train group | HDDC3 | 0.874 | 0.32 | 0.740-0.979 | 100.0 | 68.4 | < 0.001 |
|  | SDC2 | 0.881 | 0.34 | 0.758-0.972 | 93.3 | 68.4 | < 0.001 |
|  | HDDC3+SDC2 | 0.933 | 0.37 | 0.839-1.000 | 94.7 | 86.7 | < 0.001 |
| Test group | HDDC3 | 0.918 | 0.15 | 0.735-1.000 | 85.7 | 85.7 | 0.009 |
|  | SDC2 | 0.816 | 0.10 | 0.531-1.000 | 71.4 | 100.0 | 0.048 |
|  | HDDC3+SDC2 | 0.959 | 0.64 | 0.861-1.000 | 85.7 | 100.0 | 0.004 |
